# Individual and Additive Effects of Acute Intake of New Zealand Blackcurrant Powder and Caffeine on High-Intensity Intermittent Running Performance

**DOI:** 10.3390/nu18183063

**Published:** 2026-09-19

**Authors:** Krutika Nanavati, Kaio Fernando Vitzel, Kay Rutherfurd-Markwick, Roger Hurst, Ajmol Ali

**Affiliations:** 1 Eastern Institute of Technology, Auckland 1010, New Zealand; knanavati@eit.ac.nz; 2 School of Sport, Exercise, and Nutrition, Massey University, Auckland 0632, New Zealand; 3 School of Health Sciences, Massey University, Auckland 0632, New Zealand; k.vitzel@massey.ac.nz (K.F.V.); k.j.rutherfurd@massey.ac.nz (K.R.-M.); 4 Ag Emissions Centre, Bioeconomy Science Institute, Palmerston North 4442, New Zealand; hurstroger1@gmail.com; 5 School of Sport, Exercise and Health Sciences, Loughborough University, Loughborough LE11 3TU, UK

**Keywords:** polyphenols, repeated sprints, sports, nutrition

## Abstract

Background/Objectives: Consumption of New Zealand blackcurrant extract for 7 days has shown to reduce slowing of sprint speed in recreationally active males. Acute caffeine consumption has shown to improve repeated-sprint performance in a fatigued state. Thus, combined intake of New Zealand blackcurrants and caffeine may provide an additive or a synergistic effect to improve repeated-sprint performance, especially in fatigued individuals. Our study investigated the individual and additive effect of acute intake of New Zealand blackcurrant powder and caffeine on high-intensity intermittent running performance. Methods: Fourteen recreationally active males participated in a double-blind, randomised controlled crossover trial consisting of four experimental arms: placebo (PLA), New Zealand blackcurrant powder (NZBP), caffeine (CAFF), and NZBP + caffeine (NZBP + CAFF). Participants reported to the laboratory fasted and consumed a standardised breakfast with the study intervention 1 h before the exercise trial. Speed and distance covered for walking, sprinting, running, and jogging were assessed using the modified Loughborough Intermittent Shuttle Test (m-LIST). Blood lactate and free fatty acids (FFAs) levels, and perceptual measures (feeling scale, felt arousal scale and ratings of perceived exertion) were also assessed. Results: Caffeine intake increased average sprint speed (*p* = 0.045, η^2^ = 0.345) and reduced sprint time (*p* = 0.030, η^2^ = 0.463) during the m-LIST protocol. Ratings of perceived exertion were lower with caffeine intake (*p* = 0.014, η^2^ = 0.381), while blood lactate concentration was higher (*p* = 0.001, η^2^ = 0.761). Serum free fatty acids concentrations increased over time (*p* < 0.001, η^2^ = 0.900), with a significant NZBP × time interaction (*p* = 0.005, η^2^ = 0.433). No significant effects of NZBP alone or caffeine × NZBP interactions were observed for sprint performance outcomes. Conclusions: Acute NZBP did not improve repeated-sprint performance, either alone or when combined with caffeine. In contrast, caffeine-containing treatments were associated with improved sprint performance and lower perceived exertion. These findings suggest that caffeine, rather than NZBP, was responsible for the observed ergogenic responses, with no evidence of an additive benefit from acute combined supplementation.

## 1. Introduction

High-intensity intermittent running is essential for success in field-based sports such as football, field hockey, and rugby. This type of exercise consists of alternating periods of short-duration sprinting and brief active recovery periods [1]. In football, sprinting and high-speed running demands have increased substantially over recent years, and these activities now contribute meaningfully to the total distance covered during match play [2]. Linear advancing movements, including sprinting by the goal scorer or assisting player, are also among the most frequent actions preceding goals [3,4]. Therefore, the ability to attenuate fatigue and maintain repeated-sprint performance, particularly during the final stages of match play when goal scoring may be more frequent [5], may affect the outcome of the game.

Fatigue during repeated-sprint exercise typically develops rapidly after the first sprint [6]. This decline in performance is multifactorial and may involve neural factors, such as inadequate motor command from the motor cortex, as well as muscular factors, including the accumulation of metabolites such as inorganic phosphate, hydrogen ions, and adenosine diphosphate within muscle fibres [7]. During high-intensity intermittent running, fatigue may also be influenced by limitations in energy supply, including phosphocreatine hydrolysis, anaerobic glycolysis, and oxidative metabolism, as well as reduced muscle excitability associated with the accumulation of interstitial potassium ions [7]. Nutritional strategies that target fatigue-related mechanisms may therefore be useful for supporting repeated-sprint performance during intermittent field-sport activity.

Caffeine is one ergogenic aid that has been shown to improve repeated-sprint exercise performance. Caffeine supplementation has been reported to improve multiple-sprint running performance [8]. In addition, caffeine intake of 3.7 mg/kg, when combined with a 6% carbohydrate–electrolyte solution, decreased the slowing of repeated sprints during the later stages of a modified Loughborough Intermittent Shuttle Test protocol [9]. Caffeine acts through both peripheral and central mechanisms [10], including binding to adenosine receptors and blocking their inhibitory neurophysiological actions, thereby exerting a stimulatory effect on the central nervous system [11]. These effects may be particularly relevant during fatiguing intermittent exercise, where perceived exertion and the ability to sustain high-intensity efforts become progressively limiting.

New Zealand blackcurrant has also been investigated as a potential ergogenic aid for intermittent running performance. Consuming 300 mg/day of New Zealand blackcurrant extract, containing 105 mg of anthocyanins, for 7 days, with the final dose consumed 3 h before exercise, has been shown to reduce the slowing of the fastest sprint between blocks 1 and 5 during part A of the Loughborough Intermittent Shuttle Test (LIST) protocol and to increase time to exhaustion in some participants during part B [12]. The mechanism by which New Zealand blackcurrant intake may influence sport performance is not yet fully elucidated. However, a review examining dietary anthocyanins, including cherries and New Zealand blackcurrant, suggested that performance-related effects may be associated with the vasoactive properties of anthocyanins [13,14].

Training for field sports that involve high-intensity intermittent running (such as rugby) often takes place less than 48 h after match play, and athletes may be expected to train for two or more consecutive days within a week [15]. Excessive training with inadequate recovery can lead to accumulated fatigue, compromise neuromuscular performance [16,17,18], and contribute to under-performance on match day [19,20]. Under these conditions, combining ergogenic aids with different mechanisms of action may provide additive or synergistic effects. Caffeine may support performance through central nervous system stimulation [21], whereas New Zealand blackcurrant may exert effects through anthocyanin-related vasoactive properties [22]. Therefore, combined intake of caffeine and New Zealand blackcurrant could potentially improve high-intensity intermittent running performance, particularly when individuals are fatigued.

Athletes commonly consume multiple ergogenic aids concurrently in an attempt to optimise training and competition performance. However, despite the widespread use of supplement combinations in practice, relatively few studies have examined the simultaneous use of multiple ergogenic aids and their potential interactions. Burke and Peeling [23] noted that the opportunity for supplements to exert additive, neutral, or counteractive effects when used together has rarely been investigated, despite representing a common real-world supplementation strategy among athletes. Therefore, examining the combined effects of New Zealand blackcurrant and caffeine has both scientific and practical relevance and may provide insights that are more applicable to athlete practice than studies investigating these supplements in isolation.

The aim of the present study was to examine the individual and additive effects of acute intake of New Zealand blackcurrant powder (NZBP) and caffeine on sprint performance using a modified LIST protocol (m-LIST). The m-LIST protocol assesses speed, distance covered, sprint time, and reaction time across six blocks and includes a self-paced phase (blocks 5 and 6) designed to simulate the final stages of match play, when fatigue is most evident [24]. We hypothesised that a single dose of NZBP combined with caffeine, consumed in a fatigued state, would reduce the slowing of sprint performance during blocks 5 and 6 of the m-LIST protocol. To induce fatigue before the running trial, participants completed a 90 min muscle glycogen-depletion cycling protocol on the day before each experimental trial.

## 2. Materials and Methods

### 2.1. Experimental Design

This study used a double-blind, randomised, placebo-controlled crossover design to examine the individual and additive effects of acute New Zealand blackcurrant powder (NZBP) and caffeine intake on high-intensity intermittent running performance. Participants completed four experimental trials, separated by a minimum 7-day wash-out period, in which they consumed placebo, NZBP, caffeine, or NZBP combined with caffeine. The order of study drink at each trial was randomised for each participant. Each trial was conducted over two days: a 90 min glycogen-depletion cycling protocol was completed on day 1 to induce prior-exercise fatigue, and the m-LIST protocol was completed the following morning after an overnight fast, standardised breakfast, and study drink intake. The primary outcomes were sprint performance measures during the m-LIST, including sprint speed, sprint time, reaction time, movement time, and distance covered. Secondary outcomes included physiological and perceptual measures, including blood lactate, serum free fatty acids (FFA), cardiovascular responses, feeling scale (FS), felt arousal scale (FAS), and ratings of perceived exertion (RPE). The study was approved by the Massey University Human Ethics Southern A Committee (Ohu Matatika 1; approval number 21/09, 22 March 2021), registered with the Australia New Zealand Clinical Trials Registry (ACTRN12621001394831), and all participants provided written informed consent before participation.

### 2.2. Sample Size Determination

To determine the necessary sample size, a power analysis using G-Power 3.1.9.7 was conducted, focusing on the primary outcome measure of sprint performance. Comparing caffeine and placebo trials from previous studies involving well-trained male football players [9] revealed a 4.5% improvement in mean 15 m sprint performance during blocks 5 and 6 of LIST. Utilising G*Power (Version 3.1.9.7) with an alpha level of 0.05 and a power of 0.80, based on data from the study by Gant et al. (2010) [9], our estimated sample size was 12 participants. To account for any dropouts we recruited 14 participants for this study.

### 2.3. Participants

Fourteen healthy male participants were recruited from local football clubs around Auckland, New Zealand. Participants’ characteristics were: age 30 ± 9 years, height 173 ± 23 cm, body mass 77 ± 8 kg, and $\dot{V}O$_2_max 48.8 ± 4.2 mL·kg^−1^·min^−1^. Participants were recreationally active with experience in team sports with high-intensity intermittent running, but were not familiar with cycling. This study was approved by the Massey University Human Ethics Southern A Committee (Ohu Matatika 1) (approval number 21/09, 22 March 2021) and registered with the Australia New Zealand Clinical Trials Registry (ACTRN12621001394831, 15 October 2021). The participants provided written informed consent before their familiarisation visit and completed a medical history questionnaire.

### 2.4. Study Design

**Figure 1:**
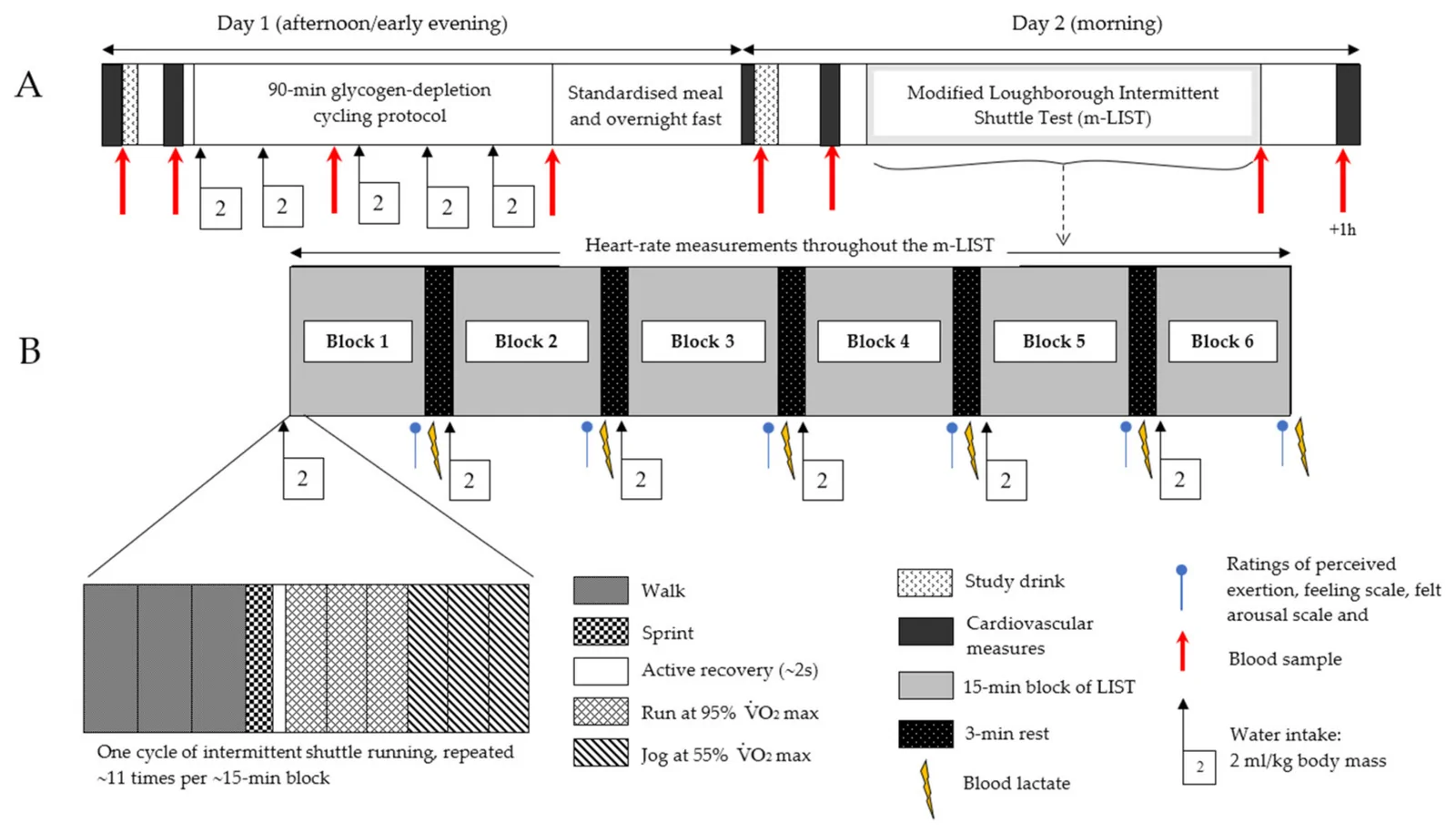
Schematic representation of (**A**) the overall experimental protocol and (**B**) the Loughborough Intermittent Shuttle Running Test.

Participants attended an initial two-part familiarisation visit for the assessment of $\dot{V}O$_2_max, and then reported to the laboratory for 4 main trials, with each trial taking place over two days (Figure 1). On day 1 of the trial, participants reported to the laboratory in the afternoon/early evening to participate in the glycogen-depletion fatiguing cycling protocol and then the following morning on day 2 for the running trial (m-LIST). The study was a double-blind, randomised, crossover trial (Latin square design) and was separated by a minimum of 7 days wash-out period. The four running trials tested four study treatments: placebo (PLA), in which participants consumed placebo with 0 mg caffeine on both day 1 and day 2; caffeine (CAFF), in which participants consumed placebo with 0 mg caffeine on day 1 and placebo with 240 mg caffeine on day 2; New Zealand blackcurrant powder (NZBP), in which participants consumed NZBP with 0 mg caffeine on both day 1 and day 2; and combined New Zealand blackcurrant powder plus caffeine (NZBP + CAFF), in which participants consumed NZBP with 0 mg caffeine on day 1 and NZBP with 240 mg caffeine on day 2. The data were collected over a period of 18 months from January 2022 to June 2023.

### 2.5. Familiarisation Visit

Participants were asked to report to the laboratory in comfortable athletic wear and running shoes and were later taken to the Massey University Recreation Centre, Albany Campus, Auckland for their familiarisation visit. The visit was divided into three parts: (i) maximal multistage 20 m shuttle run test (‘beep’ test) to estimate their $\dot{V}O$_2_max [25], (ii) undertaking the m-LIST protocol for 30 min to allow adequate understanding of the running patterns and the experimental procedures [24], and (iii) an incremental exercise test (ramp test) on a stationary cycle ergometer (Velotron Pro cycle ergometer, Racermate Inc., Seattle, WA, USA) to estimate maximum power output for the muscle glycogen-depletion protocol for day 1 of the study [26]. The value obtained from the $\dot{V}O$_2_max test was used to establish running speeds for the m-LIST protocol.

### 2.6. Glycogen-Depletion Fatiguing Cycling Protocol

Participants engaged in ~90 min of moderate-intensity intermittent cycling exercise on a stationary cycle ergometer (Velotron Pro cycle ergometer, Racermate Inc., Seattle, WA, USA) between 3 and 7 pm on the day before the m-LIST protocol to reduce glycogen stores and induce prior-exercise fatigue [27]. This approach was based on previous evidence showing that exhaustive high-intensity cycling produces substantial glycogen breakdown in both type I and type II muscle fibres, with higher depletion rates at greater exercise intensities [27]. Thus, we incorporated a ~90 min moderate-intensity intermittent cycling protocol to simulate training conditions that may lead to accumulated fatigue in athletes undertaking regular training. The protocol consisted of 30 min of cycling at 60% of the participant’s maximum power output, followed by a 2 min rest period and three 50 s cycling sprints at double the resistive load, separated by 2 min recovery periods. Following a further 2 min rest period, participants completed an additional 45 min of cycling at 60% of maximum power output to further reduce glycogen stores. Based on previously published validation data, this protocol has been shown to reduce muscle glycogen concentrations from resting values of approximately 110–170 mmol glycosyl units·kg^−1^ wet wt to ~13 mmol glycosyl units·kg^−1^ wet wt [28], representing an estimated 88–92% reduction in muscle glycogen content prior to the overnight fast. The maximum power output was established during an incremental exercise test (ramp test) on a stationary cycle ergometer (Velotron Pro cycle ergometer, Racermate Inc., Seattle, WA, USA) during the familiarisation visit [26]. Participants were allowed to reduce the intensity to a minimum of 110 W if they were unable to maintain their prescribed workload of 60% maximum power output [27]. Participants were provided with 2 mL·kg^−1^ body mass of water before exercise and after every 15 min of exercise to minimise dehydration.

### 2.7. Dietary Standardisation

Participants were asked to refrain from consuming alcohol and foods containing caffeine 48 h before their cycling protocol and till the completion of their m-LIST protocol. Participants were also asked to refrain from consuming high amounts of nutritional polyphenolic compounds and antioxidants for 48 h before their cycling protocol. This dietary restriction was implemented to minimise the potential influence of background dietary polyphenol intake on the study outcomes. Since anthocyanins are the primary bioactive compounds in New Zealand blackcurrant and may influence vascular function, substrate metabolism, and exercise performance, reducing consumption of other polyphenol-rich foods helped minimise potential confounding effects and allowed a clearer evaluation of the study intervention.

They were provided with a standardised dinner the day before the trial and breakfast on the day of the trial. Dinner included beef lasagna, garlic bread, salad greens, and a banana at a serving size of 14.84 kJ/kg (for example, a participant weighing 70 kg would require ~2930 kJ for dinner and this energy would come from 350 g beef lasagna, 70 g of garlic bread, 110 g banana, and 50 g of salad greens). The breakfast consisted of 50 g of muesli, 100 g of yoghurt, and 30 mL of milk and provided about ~1770 kJ of energy, 30 g of carbohydrate, 15 g of protein, and 10 g of fat.

### 2.8. Experimental Procedures

For the m-LIST protocol, participants reported to the lab early in the morning after completing at least an 8 h fast and were asked to provide a mid-stream urine sample to determine hydration status based on urine specific gravity (USG) (Atago 2773 MASTER-SUR/NM Clinical Refractometer, Atago Co., LTD., Tokyo, Japan). If found to be dehydrated (i.e., USG > 1.020) participants consumed 200 mL of water before their cardiovascular assessment (and consumed 200 mL of water at every exercise visit to maintain consistency in testing procedure). The USG test was followed by assessment of cardiovascular measures and consumption of the study drink along with breakfast. Cardiovascular measures were reassessed one hour later and this was followed by a standardised 10 min warm-up and the m-LIST protocol. Ambient temperature (19.0 to 21.0 °C) and humidity (65 to 75%) were similar between experimental trials.

### 2.9. Cardiovascular Measures

Participants were asked to remain in a supine position for assessment of blood pressure and heart rate (Omron, Healthcare CO., Ltd.; Kyoto, Japan) and stroke volume and systemic vascular resistance (Uscom 1A, Uscom Ltd., Sydney, Australia). The assessment was carried out at baseline, 1 h after study drinks were consumed, and 1 h after completion of exercise. Heart rate was also assessed during the m-LIST protocol using a wearable heart rate monitor (Polar M200, Polar, Kempele, Finland). Results for cardiovascular measures are presented in Appendix A.1.

### 2.10. Intervention

After completing baseline cardiovascular measures, participants consumed either 24 g of NZBP (containing 240 mg of anthocyanins) with or without 240 mg of caffeine or placebo (PLA) mixed with 200 mL of water. The PLA contained the same amount of maltodextrin as the NZBP with added fructose, glucose, and colour to match the nutritional value and appearance of the NZBP (Table 1). Relative dose of caffeine for our study participants was 3.15 ± 0.38 mg/kg body weight (95% CI [2.9, 3.4]). All four powders were supplied by the study sponsor.

### 2.11. Modified Loughborough Intermittent Shuttle Test (m-LIST)

The m-LIST protocol is a validated and reliable protocol that consists of 6 × 15 min blocks divided into four blocks of “prescribed-pace” activity (blocks 1–4; participants exercise based on audible signals) followed by two blocks of “self-paced” exercise (blocks 5–6; no audible signals) with a 3 min rest period between each block [24]. Each block consisted of repeated sequences called cycles of 3 × 20 m walks at 5.4 km/h, 1 × 15 m sprint, 3 × 20 m run at a speed equivalent to 95% of $\dot{V}O$_2_max and 3 × 20 m jog at a speed equivalent to 55% $\dot{V}O$_2_max. A proprietary mobile application (app) produced audio signals guiding participants’ speeds during each block. Sprint times were measured using infrared photoelectric cells present in the sprint gates, transmitted wirelessly to the app. Participants completed ~11 cycles per block from blocks 1 to 4 as they were “prescribed-pace” activity, and they received audio signals for repeated cycles. However, for “self-paced” blocks 5 and 6, participants continued the repeated sequence until they received a signal for cessation of exercise at the end of 15 min for both the blocks. Participants were instructed to replicate intensities and exercise patterns observed during the prescribed-pace blocks (for more detailed information see Ali, Foskett and Gant [24]). Participants consumed water at a rate of 2 mL/kg BM after warm-up, followed by 2 mL/kg BM after every block during blocks 1 to 5. The m-LIST protocol assessed measures of speed, distance covered, sprint time, reaction time, and movement (sum of sprint and reaction time) for sprint performance from blocks 1 to 6, and speed and distance covered for walking, jogging, and running during blocks 5 to 6 [24]. Before starting the m-LIST protocol participants underwent a standardised 10 min warm-up consisting of a total of 640 m of jogging, running, and sprinting as well as dynamic and static flexibility exercises of lower limbs and core muscles.

### 2.12. Perceptual Measures

Perceptual measures were assessed using the FS, FAS, and RPE before, during, and after the m-LIST protocol. The FS is a 11-point scale ranging from very bad (−5), bad (−3), fairly bad (−1), neutral (0) to fairly good (+1), and good (+3), and very good (+5) [29]. The FAS measures perceived activation along a 6-point scale ranging from low arousal (1 point) indicating boredom or calmness to high arousal (6 points) suggested by anger or anxiety [30] (see Appendix A.2 for results). The RPE is a 15-point scale to assess subject level of exertion intensity. The score ranges from 6 to 20, with 6 being no exertion at all to 20 being maximum exertion perceived [31].

### 2.13. Blood Sampling and Lactate Measurement

Blood samples were collected by venepuncture from a vein within the antecubital area. Samples were taken at baseline, 1 h after consumption of the study drinks, immediately after exercise, and 1 h after exercise. Eight millilitres of blood was collected into a heparin collection tube (BD Vacutainers^®^, Franklin Lakes, NJ, USA) and the samples were centrifuged for 10 min at 1250× *g* (MF 50, Hanil Science Industrial Co., Ltd., Incheon, Republic of Korea) to obtain plasma. Two millilitres of blood sample was collected into a serum collection tube (BD Vacutainers^®^, Franklin Lakes, NJ, USA) and the samples were allowed to clot at room temperature for 20 min before being centrifuged for 10 min at 1250× *g*). Plasma and serum samples were aliquoted into 1.5 mL microtubes and later stored at −80 °C (Thermo Fisher Scientific TSU TSU600D −80 °C Upright ULT Ultra-Low Temperature Freezer, Thermo Fisher Scientific, Asheville, NC, USA) for analysis of caffeine metabolites and FFA respectively. Blood lactate was assessed from capillary blood (finger prick) using handheld lactate meter (Lactate Pro, Arkray, Shiga, Japan) after completion of each block of the m-LIST protocol.

### 2.14. Blood Analysis

Plasma caffeine and caffeine metabolites (paraxanthine and theophylline) were analysed by reversed-phase HPLC using a LC-20 liquid chromatograph equipped with an SPD-M20A photodiode array detector (Shimadzu Inc., Kyoto, Japan) [32]. Frozen plasma samples were thawed and deproteinised by adding 400 μL of 0.8 M perchloric acid to 400 μL of the sample. Samples were vortexed for 10 s and then centrifuged for 10 min at 9900× *g*. The supernatant was transferred to a glass vial and used for reversed-phase HPLC. Detailed results for plasma caffeine and caffeine metabolite concentrations are presented in Appendix A.3.

Serum FFA were analysed using the Randox non-esterified fatty acids kit (FA 115, Randox Laboratories Limited, Crumlin, Northern Ireland) according to the manufacturer’s instructions using a clinical chemistry analyser (RX Daytona plus, Randox Laboratories Limited, Crumlin, Northern Ireland) at the Nutrition Laboratory, School of Food and Advanced Technology, Massey University, Palmerston North, New Zealand.

### 2.15. Statistical Analysis

Data were analysed using factorial repeated-measures analysis of variance (ANOVA) in IBM SPSS Statistics (version 28.0.1; IBM, Armonk, NY, USA). Caffeine (absent vs. present), NZBP (absent vs. present), and time were included as within-participant factors for all outcomes to assess their individual and combined effects across repeated measurements. The models tested the main effects of caffeine, NZBP, and time, together with the caffeine × NZBP, caffeine × time, NZBP × time, and caffeine × NZBP × time interactions. To account for violations of sphericity, degrees of freedom were adjusted according to the Greenhouse–Geisser epsilon (ε): the Huynh–Feldt correction was applied when ε > 0.75, and the Greenhouse–Geisser correction was applied when ε < 0.75. Where a significant main effect or interaction was identified, Bonferroni-adjusted pair-wise comparisons with 95% confidence intervals were used to examine specific differences. Statistical significance was accepted at *p* < 0.05. Partial eta squared (η^2^) was calculated as the effect-size measure and interpreted as small (0.01), medium (0.06), or large (0.14) [33]. Data normality was assessed using the Shapiro–Wilk test, and all variables were considered normally distributed. Data are presented as mean ± SD.

## 3. Results

### 3.1. Sprint Performance During Modified Loughborough Intermittent Shuttle Test

Caffeine increased average sprint speed across blocks 1 to 6 of the m-LIST (mean difference = 0.45 km/h, 95% CI [0.01, 0.89], *p* = 0.045, ηp^2^ = 0.345). Average sprint speed also decreased over time (*p* = 0.032, ηp^2^ = 0.341). There was no caffeine × time, NZBP × time, or caffeine × NZBP × time interaction (*p* = 0.692, 0.152, and 0.244, respectively) (Table 2).

Distance covered during the prescribed-pace blocks 1 to 4 was constant for all four treatments (165 m; 11 × 15 m sprints per block) and decreased during the self-paced blocks 5 and 6 (block 5, PLA: 150 ± 10 m, NZBP: 150 ± 10 m, CAFF: 150 ± 10 m, and NZBP + CAFF: 150 ± 10 m; and block 6, PLA: 150 ± 10 m, NZBP: 150 ± 10 m, CAFF: 150 ± 10 m, and NZBP + CAFF: 160 ± 10 m; *p* = 0.001, η^2^ = 0.662). No caffeine × time, NZBP × time, or caffeine × NZBP × time interaction was observed for distance covered.

Caffeine intake reduced sprint time (mean difference = −0.18 s, 95% CI [−0.34, −0.02]; *p* = 0.030, η^2^ = 0.463), indicating faster sprint performance. Sprint time did not change over time, whereas reaction time and movement time increased across blocks 1 to 6 (*p* < 0.001, η^2^ = 0.927 and *p* < 0.001, η^2^ = 0.842, respectively) indicating slower responses as the protocol progressed. No caffeine × time, NZBP × time, or caffeine × NZBP × time interaction was observed for sprint time, reaction time, or movement time (Table 2). Furthermore, no trial-order effect was observed for any m-LIST variable.

### 3.2. Perceptual Measures

Ratings of perceived exertion were lower with caffeine intake (mean difference = −0.74, 95% CI [−1.30, −0.17]; *p* = 0.014, η^2^ = 0.381) and increased over time (*p* < 0.001, η^2^ = 0.688). No caffeine × time, NZBP × time, or caffeine × NZBP × time interaction was observed for RPE (Table 3). Detailed descriptive and statistical results for the feeling scale and FAS are presented in Appendix A.2. (Table A3).

### 3.3. Blood Lactate

Blood lactate concentration was higher with caffeine intake (mean difference = 1.3 mmol/L, 95% CI [0.8, 1.9]; *p* = 0.001, η^2^ = 0.761). There was no effect of time (*p* = 0.420, η^2^ = 0.087), and no caffeine × time, NZBP × time, or caffeine × NZBP × time interaction was observed for blood lactate (Table 3).

### 3.4. Serum FFA

**Figure 2:**
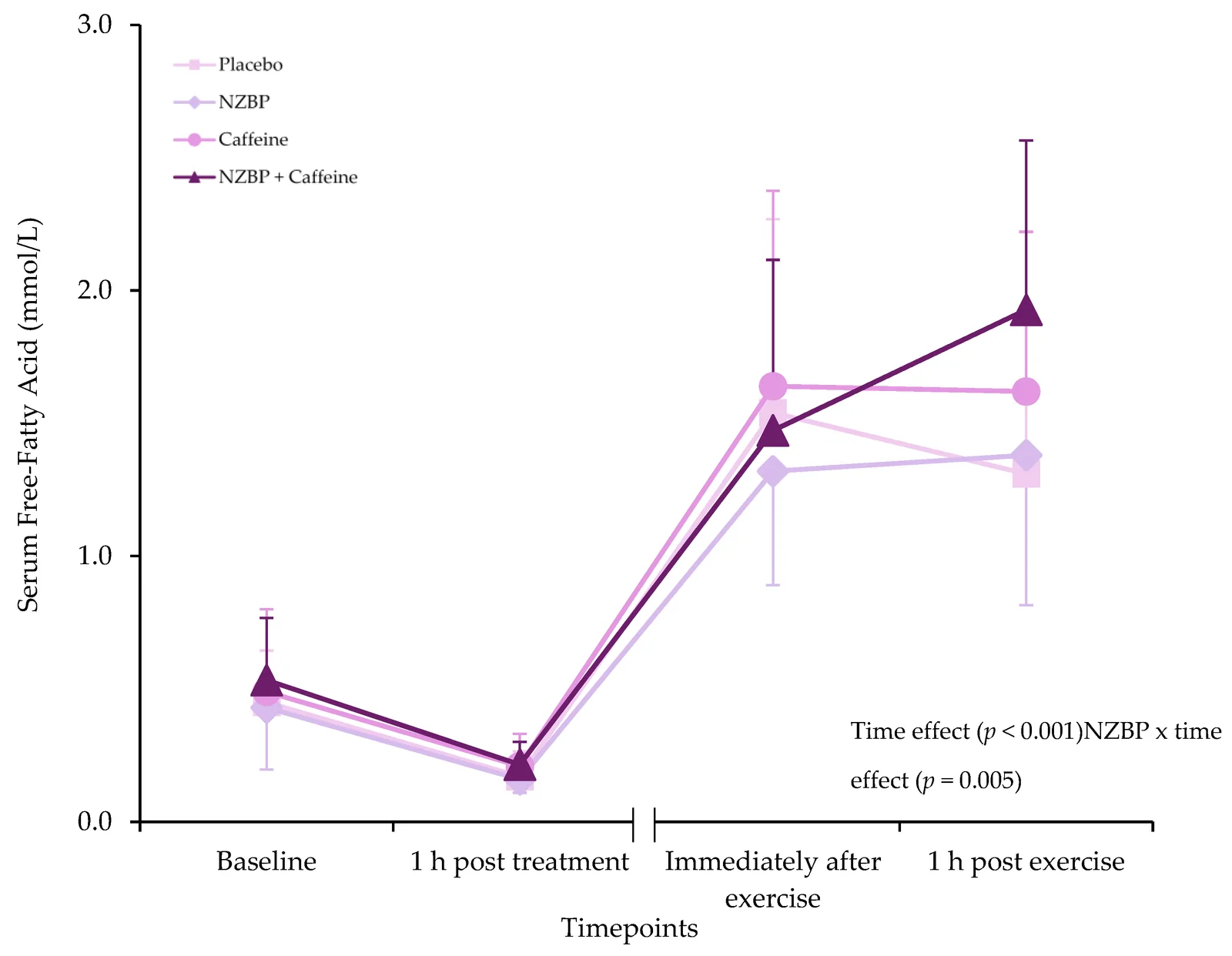
Serum free fatty acids concentration (mean ± SD) for placebo (PLA), caffeine (CAFF), NZBP, and NZBP + caffeine (NZBP + CAFF) trials at baseline, 1 h after consuming the study drink, immediately after exercise, and 1 h after exercise.

Serum FFA concentrations increased over time (*p* < 0.001, η^2^ = 0.900) and an interaction effect was observed for NZBP × time (*p* = 0.005, η^2^ = 0.433). Immediately after exercise, NZBP had lower serum FFA concentration (mean difference = −0.33 mmol/L, 95% CI [−0.56, −0.10], *p* = 0.009). There was no effect of caffeine, NZBP, caffeine × NZBP, caffeine × time, or caffeine × NZBP × time interaction (Figure 2; Table 4). Furthermore, there was a trial-order effect (two-way repeated measures ANOVA; *p* = 0.007, η^2^ = 0.326). Bonferroni-adjusted pairwise comparisons showed that, immediately after exercise, serum FFA concentration was lower when NZBP was present than when NZBP was absent (mean difference = −0.33 mmol/L, 95% CI [−0.56, −0.10], *p* = 0.009).

## 4. Discussion

This study examined the individual and combined effects of acute New Zealand blackcurrant powder and caffeine intake on high-intensity intermittent running performance in a fatigued state. Overall, acute NZBP did not improve repeated-sprint performance, either alone or when combined with caffeine. However, caffeine-containing treatments appeared to better sustain sprint performance and reduce perceived exertion, suggesting that caffeine may have had a greater influence than NZBP under the present conditions.

The decline in sprint speed across the m-LIST indicates that the protocol successfully induced fatigue, but treatment-related performance differences were modest. Caffeine had a significant effect on average sprint speed and sprint time, whereas NZBP and the caffeine × NZBP interaction were not significant. These findings suggest that the favourable responses observed with the NZBP + CAFF treatment were primarily attributable to caffeine rather than an additive effect of NZBP. This finding is also consistent with Best et al. [34], who reported no improvement in repeated-sprint ability following New Zealand blackcurrant extract supplementation. Similarly, Paton et al. [35] reported that acute New Zealand blackcurrant extract did not provide additional performance benefits when combined with caffeine during repeated high-intensity cycling. Although participants in the present study completed a glycogen-depletion protocol before exercise, those in Paton et al. [35] were not assessed in a fatigued state; both studies indicate that acute NZBP supplementation does not enhance the ergogenic effects of caffeine during repeated high-intensity exercise.

The changes in sprint performance associated with caffeine intake may be partly explained by its central effects. Caffeine acts primarily as an adenosine receptor antagonist, increasing central nervous system stimulation and reducing perceptions of effort [10,11]. In the present study, RPE increased over time, and caffeine had a significant effect on RPE, whereas NZBP and the caffeine × NZBP interaction were not significant. This finding is consistent with a previous work showing improved perceptual responses during intermittent exercise after caffeine intake [36] and may partly explain the significant caffeine effects observed for average sprint speed and sprint time.

The absence of a clear additive or synergistic effect of NZBP + CAFF may relate to the acute dosing strategy used in the present study. Previous studies reporting benefits of New Zealand blackcurrant have often used multi-day supplementation [12,37], which may allow greater accumulation of anthocyanin metabolites or more sustained effects on vascular and metabolic pathways. Although anthocyanins have been proposed to enhance exercise performance through vasoactive effects, such as improved endothelial function, peripheral blood flow, and oxygen delivery [13,14,22], these mechanisms did not appear to be a limiting factor for repeated-sprint performance [38,39]. This is supported by the absence of clear treatment effects on cardiovascular measures and the lack of a significant main effect of NZBP on sprint performance. In addition, the standardised breakfast consumed with the intervention may have influenced the absorption and availability of anthocyanins and caffeine, potentially limiting the timing or magnitude of any combined effect during the m-LIST. It is also possible that caffeine was the dominant ergogenic stimulus in the combined treatment, leaving limited scope for an additional measurable benefit from NZBP. Therefore, the absence of significant caffeine × NZBP interactions across the principal outcomes does not support a synergistic effect of acute combined intake.

At present, limited evidence is available regarding the acute effects of New Zealand blackcurrant on running performance. A single dose of New Zealand blackcurrant extract has been shown to improve 5 km time-trial performance in trained runners [40], whereas other studies reporting benefits for intermittent running have used 7-day supplementation periods [12,37]. For example, Willems et al. [12] reported less slowing of sprint performance during the LIST protocol, while Perkins et al. [37] reported increased distance covered during an incremental high-intensity intermittent running (adapted from National Institute of Education, Singapore intermittent high-intensity test) following multi-day New Zealand blackcurrant extract intake. Therefore, it is possible that the ergogenic effects of New Zealand blackcurrant may be more apparent during exercise capacity or time-trial tasks, or after repeated supplementation, rather than during an acute repeated-sprint protocol such as the m-LIST.

The metabolic responses indicate that caffeine influenced blood lactate concentrations, although these findings should be interpreted cautiously. Blood lactate showed a significant effect of caffeine, whereas NZBP and the caffeine × NZBP interaction were not significant. Blood lactate concentrations increase during repeated-sprint exercise as muscles rely heavily on anaerobic glycolysis [1], and higher lactate responses have been reported following caffeine intake during high-intensity exercise [41,42]. This caffeine-related metabolic response occurred alongside significant caffeine effects on RPE, average sprint speed, and sprint time. However, the absence of significant time interactions indicates that the pattern of blood lactate responses across the m-LIST did not differ according to caffeine or NZBP intake.

Serum FFA concentrations increased over time during the m-LIST. A significant NZBP × time interaction pointed out to a lower increase in serum FFA concentration immediately after exercise with NZBP intake. However, there was no significant effect of caffeine intake. Previous studies have reported that New Zealand blackcurrant intake may influence fat oxidation during exercise [43], while caffeine has also been associated with altered substrate metabolism during exercise [11]. Serum FFA concentrations alone do not confirm an effect on fat oxidation, and because substrate metabolism and intramuscular phosphocreatine recovery were not directly measured, the relevance of the NZBP × time interaction to repeated-sprint performance remains uncertain.

From a practical perspective, the moderate-to-large effect sizes observed for several outcomes suggest that some treatment-related differences may still be relevant for athletes participating in intermittent sports, even when statistical significance was not reached. In field-based sports, small improvements in sprint speed, sprint time, or the ability to tolerate perceived exertion may be meaningful, particularly during repeated high-intensity efforts late in training or match play [1,24]. However, these findings should be interpreted cautiously because the study involved recreationally active males, the sample size was modest, and most treatment effects were not consistently accompanied by statistically significant post hoc comparisons. Thus, while caffeine-containing treatments appeared to show the most promising practical responses, the present findings do not provide sufficient evidence to recommend acute NZBP + CAFF intake as a clearly superior strategy for intermittent-sport performance.

Future studies should investigate whether longer supplementation periods with New Zealand blackcurrant, alone and combined with caffeine, produce clearer effects in trained athletes. Further work should also assess plasma anthocyanins and anthocyanidins (e.g., cyanidin and delphinidin), caffeine metabolites, plasma glycerol, and other markers of substrate metabolism to clarify the physiological mechanisms underlying any potential ergogenic effect. In addition, future studies should examine whether the vasoactive properties of anthocyanins contribute meaningfully to performance under conditions where oxygen delivery or vascular function may be more limiting.

## 5. Limitations and Future Directions

The Latin square design with four arms used in this study was challenging due to the heavy burden it placed on participants and also due to injuries and unavailability. This study was also affected in a similar way by the COVID-19 pandemic. This resulted in incomplete trials, and challenges to recruitment of well-trained participants (who could not dedicate time for the trials due to their other heavy training load commitments). In addition, the power analysis for sample size was based on data from well-trained male football players, which may not translate directly to recreationally active males exercising in a fatigued state. Although the study exceeded the target sample size determined from the a priori power analysis (14 participants completed the study compared with 12 required), the magnitude and variability of response in recreationally active individuals may differ from those observed in trained athletes. Therefore, future studies involving larger cohorts and/or well-trained athletes may help determine whether training status influences the response to New Zealand blackcurrant and caffeine supplementation.

The present analysis also only focused on cohort-level responses and was not designed or powered to formally assess interindividual variability in response to NZBP, caffeine, or their combined intake. Future studies should use approaches that distinguish true individual responses from random within-participant variation [44].

A further limitation is that muscle glycogen-depletion or comparable pre-exercise fatigue status was not directly confirmed before the m-LIST trial, for example using muscle biopsy or another objective marker; therefore, it cannot be confirmed that participants began each exercise trial with equivalent levels of glycogen depletion or fatigue.

Another limitation is that participants were required to follow a low-polyphenol diet for 48 h before each experimental trial and until completion of the exercise protocol. While this approach was necessary to minimise potential confounding from dietary polyphenols and to better isolate the effects of the New Zealand blackcurrant intervention, it may not reflect habitual dietary practices of athletes and therefore may limit the ecological validity of the findings.

Furthermore, habitual caffeine intake was also not assessed. As habitual caffeine consumption may influence the ergogenic response to acute caffeine supplementation, it is unclear whether differences in caffeine habituation contributed to the observed responses. Future studies should assess habitual caffeine intake and explore its potential influence on the effectiveness of caffeine and New Zealand blackcurrant supplementation. Furthermore, a fixed caffeine dose of 240 mg was used in this study because caffeine was provided as part of a commercially formulated supplement. Consequently, relative caffeine intake varied according to body mass. Although the mean relative dose was within the range commonly reported to be ergogenic (~3 mg/kg body mass), variation in relative caffeine exposure between participants may have contributed to variability in the physiological and performance responses observed.

It is also possible that consumption of the standardised breakfast influenced the absorption and availability of New Zealand blackcurrant anthocyanins in this cohort. However, without measuring plasma anthocyanin levels, the profile of plasma anthocyanin concentrations over time remains unknown. Lastly, we only used serum FFA concentrations as an indicator of fat metabolism. Additional assessments of plasma glycerol and indirect calorimetry may have provided a more comprehensive understanding of the effects of acute New Zealand blackcurrant consumption on lipolysis and fatty acid oxidation.

The absence of a clear ergogenic effect of NZBP alone may relate to the acute dosing strategy used in this study. As previous studies have demonstrated performance benefits with multi-day supplementation protocols [12,43], the single dose used here may have been insufficient to allow anthocyanin metabolites to accumulate and exert meaningful vascular, metabolic, or performance-related effects. Consequently, acute NZBP intake may not have been sufficient to influence repeated-sprint performance in recreationally active participants exercising in a fatigued state.

Future studies should define the minimum daily consumption required for New Zealand blackcurrant to elicit an ergogenic effect and determine whether longer supplementation periods enhance or diminish these effects. Assessment of time-to-exhaustion performance may also be valuable, as no changes in speed or distance covered were observed during the 90 min m-LIST protocol. In addition, evaluation of plasma anthocyanin concentrations and cellular uptake before and following standardised meals, as well as before, during, and after exercise, may provide further insight into anthocyanin absorption, metabolism, and mechanisms of action.

## 6. Conclusions

This study was the first to examine the individual and combined effects of a single dose of New Zealand blackcurrant powder and caffeine on performance during m-LIST in fatigued recreationally active males. The findings suggest that caffeine, with and without New Zealand blackcurrant powder, may help sustain sprint speed during high-intensity intermittent running, although no significant improvements were observed in the primary performance outcomes assessed during the m-LIST. These findings also suggest that acute New Zealand blackcurrant intake may be insufficient to enhance intermittent running performance and that a longer supplementation period may be required. While caffeine consumption reduced perceived exertion, further research is needed to investigate the effects of New Zealand blackcurrant and caffeine on substrate metabolism and exercise performance in well-trained athletes and across different exercise modalities.

## Figures and Tables

**Table 1:** Nutritional content of NZBP, NZBP + CAFF, CAFF, and PLA powder (per 24 g of serving).

	PLA	CAFF	NZBP	NZBP + CAFF
Energy (kJ)	377	377	377	377
Energy (kcals)	90	90	90	90
Total carbohydrates (g)	20.6	20.6	20.6	20.6
Total added sugar (g)	20.6	20.6	9	9
Vitamin C (mg)	0	0	140	140
Blackcurrant anthocyanins (mg)	0	0	240	240
Caffeine (mg) (from green coffee beans)	0	240	0	240

PLA, placebo powder; CAFF, caffeinated placebo powder; NZBP, New Zealand blackcurrant powder; NZBP + CAFF powder, caffeinated New Zealand blackcurrant powder. Other ingredients: Maltodextrin, monk fruit extract, natural flavours (no added colours or preservatives).

**Table 2:** Sprint performance during blocks 1–6 of the modified Loughborough Intermittent Shuttle Test.

		Block 1	Block 2	Block 3	Block 4	Block 5	Block 6	Effect of Caffeine	Effect of NZBP	Effect of Caffeine × NZBP	Effect of Time	Effect of Caffeine × Time	Effect of NZBP × Time	Effect of Caffeine × NZBP × Time
Average Sprint speed (km/h)	PLA	18.1 ± 2.4	18.2 ± 2.5	17.7 ± 2.4	17.5 ± 2.2	17.0 ± 0.3	16.7 ± 3.3	***p* = 0.045** η^2^ = 0.345	*p* = 0.209 η^2^ = 0.153	*p* = 0.112 η^2^ = 0.233	***p* = 0.032** η^2^ = 0.341	*p* = 0.692 η^2^ = 0.024	*p* = 0.152 η^2^ = 0.169	*p* = 0.244 η^2^ = 0.133
	NZBP	17.8 ± 2.4	17.7 ± 2.6	17.2 ± 2.5	16.9 ± 2.4	16.4 ± 3.1	16.4 ± 3.3							
	CAFF	19.0 ± 2.2	18.7 ± 2.1	18.2 ± 2.1	18.0 ± 2.2	17.5 ± 2.8	17.3 ± 2.8							
	NZBP + CAFF	18.4 ± 2.2	18.1 ± 2.4	18.3 ± 2.4	18.0 ± 2.3	17.1 ± 3.1	17.4 ± 3.1							
Sprint time (s)	PLA	2.7 ± 0.8	2.5 ± 0.9	2.7 ± 1.0	2.9 ± 0.9	2.6 ± 1.1	3.0 ± 0.6	***p* = 0.030** η^2^ = 0.463	*p* = 0.172 η^2^ = 0.219	*p* = 0.704 η^2^ = 0.019	*p* = 0.221 η^2^ = 0.178	*p* = 0.099 η^2^ = 0.229	*p* = 0.164 η^2^ = 0.202	*p* = 0.601 η^2^ = 0.054
	NZBP	2.5 ± 1.0	2.5 ± 1.0	2.7 ± 1.1	2.8 ± 0.8	3.0 ± 0.6	2.9 ± 0.7							
	CAFF	2.3 ± 1.0	2.3 ± 1.0	2.5 ± 1.0	2.4 ± 1.0	2.2 ± 1.2	2.3 ± 1.0							
	NZBP + CAFF	2.4 ± 1.0	2.4 ± 1.0	2.5 ± 1.0	2.5 ± 1.0	2.9 ± 0.3	2.7 ± 0.5							
Reaction time (s)	PLA	1.0 ± 0.4	1.2 ± 0.6	1.1 ± 0.7	1.1 ± 0.8	2.1 ± 0.4	2.5 ± 0.7	*p* = 0.579 η^2^ = 0.054	*p* = 0.754 η^2^ = 0.018	*p* = 0.305 η^2^ = 0.173	***p* < 0.001** η^2^ = 0.927	*p* = 0.663 η^2^ = 0.068	*p* = 0.407 η^2^ = 0.139	*p* = 0.212 η^2^ = 0.226
	NZBP	1.0 ± 0.4	1.1 ± 0.4	1.1 ± 0.4	1.1 ± 0.4	2.5 ± 0.8	2.5 ± 0.8							
	CAFF	1.1 ± 0.3	1.1 ± 0.4	1.1 ± 0.4	1.2 ± 0.5	2.6 ± 1.1	2.5 ± 0.7							
	NZBP + CAFF	1.1 ± 0.3	1.2 ± 0.4	1.2 ± 0.6	1.1 ± 0.5	2.0 ± 0.7	2.2 ± 0.8							
Movement time (s)	PLA	3.6 ± 1.1	3.6 ± 1.1	3.8 ± 1.1	3.8 ± 1.2	4.3 ± 1.3	5.0 ± 0.9	*p* = 0.618 η^2^ = 0.029	*p* = 0.512 η^2^ = 0.049	*p* = 0.848 η^2^ = 0.004	***p* < 0.001** η^2^ = 0.842	*p* = 0.245 η^2^ = 0.144	*p* = 0.058 η^2^ = 0.273	*p* = 0.655 η^2^ = 0.45
	NZBP	3.6 ± 1.0	3.6 ± 1.0	3.8 ± 1.1	3.8 ± 1.2	4.8 ± 1.0	4.8 ± 1.0							
	CAFF	3.3 ± 1.3	3.3 ± 1.4	3.4 ± 1.4	3.5 ± 1.5	4.3 ± 1.3	4.2 ± 1.3							
	NZBP + CAFF	3.4 ± 1.4	3.5 ± 1.4	3.5 ± 1.5	3.5 ± 1.5	4.7 ± 0.7	4.7 ± 0.7							

PLA, placebo powder; CAFF, caffeinated placebo powder; NZBP, New Zealand blackcurrant powder; NZBP + CAFF powder, caffeinated New Zealand blackcurrant powder. Values are mean ± SD. Data were analysed using factorial repeated-measures ANOVA with caffeine, NZBP, and time as within-participant factors. Significant effect of caffeine, NZBP, and time, and the caffeine × NZBP, caffeine × time, NZBP × time, and caffeine × NZBP × time interactions were assessed. Significant effects are shown in bold.

**Table 3:** Ratings of perceived exertion and blood lactate after blocks 1 to 6 during the modified Loughborough Intermittent Shuttle Test.

		Block 1	Block 2	Block 3	Block 4	Block 5	Block 6	Effect of Caffeine	Effect of NZBP	Effect of Caffeine × NZBP	Effect of Time	Effect of Caffeine × Time	Effect of NZBP × Time	Effect of Caffeine × NZBP × Time
RPE (6 to 20)	PLA	13.9 ± 2.1	14.9 ± 1.7	15.4 ± 1.9	15.6 ± 2.1	15.6 ± 1.9	16.7 ± 1.9	***p* = 0.014** η^2^ = 0.381	*p* = 0.296 η^2^ = 0.084	*p* = 0.879 η^2^ = 0.002	***p* < 0.001** η^2^ = 0.688	*p* = 0.197 η^2^ = 0.112	*p* = 0.493 η^2^ = 0.057	*p* = 0.099 η^2^ = 0.146
	NZBP	12.6 ± 2.5	14.2 ± 2.1	15.2 ± 1.7	15.8 ± 1.6	16.0 ± 1.8	16.5 ± 1.9							
	CAFF	12.0 ± 1.8	13.8 ± 1.6	14.6 ± 1.5	15.1 ± 2.0	15.7 ± 1.7	16.4 ± 2.1							
	NZBP + CAFF	12.1 ± 1.4	13.4 ± 1.6	13.9 ± 1.7	15.1 ± 1.8	15.4 ± 1.6	16.1 ± 2.0							
Blood Lactate (mmol/L)	PLA	4.8 ± 2.5	4.7 ± 2.7	4.1 ± 1.4	5.0 ± 3.0	3.9 ± 3.2	3.7 ± 2.0	***p* = 0.001** η^2^ = 0.761	*p* = 0.695 η^2^ = 0.016	*p* = 0.473 η^2^ = 0.053	*p* = 0.420 η^2^ = 0.087	*p* = 0.269 η^2^ = 0.114	*p* = 0.610 η^2^ = 0.051	*p* = 0.874 η^2^ = 0.027
	NZBP	4.1 ± 1.7	4.1 ± 2.3	4.1 ± 2.2	4.2 ± 3.1	3.4 ± 1.7	3.2 ± 1.6							
	CAFF	5.1 ± 1.8	6.2 ± 3.0	5.0 ± 2.0	5.1 ± 2.3	4.6 ± 2.7	5.9 ± 4.5							
	NZBP + CAFF	5.1 ± 1.8	5.9 ± 2.9	6.7 ± 1.9	5.2 ± 2.1	5.0 ± 2.5	4.9 ± 1.8							

RPE, ratings for perceived exertion; PLA, placebo powder; NZBP, New Zealand blackcurrant powder; CAFF, caffeinated placebo powder; NZBP + CAFF powder, caffeinated New Zealand blackcurrant powder. Values are mean ± SD. Data were analysed using factorial repeated-measures ANOVA with caffeine, NZBP, and time as within-participant factors. Significant effect of caffeine, NZBP, and time, and the caffeine × NZBP, caffeine × time, NZBP × time, and caffeine × NZBP × time interactions were assessed. Significant effects are shown in bold.

**Table 4:** Free fatty acids concentrations at baseline, 1 h after study drink consumption, and immediately after exercise, and 1 h after exercise (modified Loughborough Intermittent Shuttle Test).

		Baseline	1 h After Study Drink	Immediately After Exercise	1 h After Exercise	Effect of Caffeine	Effect of NZBP	Effect of Caffeine × NZBP	Effect of Time	Effect of Caffeine × Time	Effect of NZBP × Time	Effect of Caffeine × NZBP × Time
Free fatty acids (mmol/L)	PLA	0.45 ± 0.19	0.17 ± 0.09	1.54± 0.73	1.31 ± 0.58	*p* = 0.086 η^2^ = 0.266	*p* = 0.275 η^2^ = 0.117	*p* = 0.860 η^2^ = 0.003	***p* < 0.001** η^2^ = 0.900	*p* = 0.064 η^2^ = 0.241	***p* = 0.005** η^2^ = 0.433	*p* = 0.960 η^2^ = 0.032
	NZBP	0.43 ± 0.23	0.16 ± 0.05	1.32 ± 0.43	1.38 ± 0.56							
	CAFF	0.49 ± 0.31	0.21 ± 0.12	1.64 ± 0.74	1.62 ± 0.60							
	NZBP + CAFF	0.53 ± 0.23	0.22 ± 0.09	1.47 ± 0.64	1.93 ± 0.64							

PLA, placebo powder; CAFF, caffeinated placebo powder; NZBP, New Zealand blackcurrant powder; NZBP + CAFF powder, caffeinated New Zealand blackcurrant powder. Values are mean ± SD. Data were analysed using factorial repeated-measures ANOVA with caffeine, NZBP, and time as within-participant factors. Significant effect of caffeine, NZBP, and time, and the caffeine × NZBP, caffeine × time, NZBP × time, and caffeine × NZBP × time interactions were assessed. Significant effects are shown in bold.

## Data Availability

Summary data are presented in this article. The raw participant-level data are available from the corresponding author upon request. The individual data for each participant is not publicly available due to ethical and privacy considerations.

## Abbreviations

The following abbreviations are used in this manuscript:

ANOVA	Analysis of variance
BM	Body mass
CAFF	Caffeine
CI	Confidence interval
FAS	Felt arousal scale
FFA	Free fatty acids
FS	Feeling scale
HPLC	High-performance liquid chromatography
HR	Heart rate
LIST	Loughborough Intermittent Shuttle Test
m-LIST	Modified Loughborough Intermittent Shuttle Test
NZBP	New Zealand blackcurrant powder
NZBP + CAFF	New Zealand blackcurrant powder combined with caffeine
PLA	Placebo
RPE	Ratings of perceived exertion
SD	Standard deviation
SVR	Systemic vascular resistance
USG	Urine specific gravity
$\dot{V}O$_2_max	Maximal oxygen uptake

## Appendix A

### Appendix A.1 Cardiovascular Measures

**Table A1:** Cardiovascular measures at baseline, 1 h after consuming the study drink, and 1 h after completing the exercise.

		Baseline	1 h After Consuming the Study Drink	1 h After Exercise	Effect of Caffeine	Effect of NZBP	Effect of Caffeine × NZBP	Effect of Time	Effect of Caffeine × Time	Effect of NZBP × Time	Effect of Caffeine × NZBP × Time
Systolic Blood Pressure (mmHg)	PLA	121 ± 5	121 ± 9	112 ± 11	*p* = 0.213 η^2^ = 0.116	*p* = 0.284 η^2^ = 0.088	*p* = 0.367 η^2^ = 0.063	***p* < 0.001** η^2^ = 0.630	***p* = 0.036** η^2^ = 0.226	*p* = 0.834 η^2^ = 0.014	*p* = 0.138 η^2^ = 0.141
	NZBP	118 ± 6	120 ± 8	111 ± 9							
	CAFF	118 ± 6	126 ± 8	113 ± 7							
	NZBP + CAFF	120 ± 7	125 ± 6	110 ± 8							
Diastolic Blood Pressure (mmHg)	PLA	73 ± 5	73 ± 5	70 ± 7	***p* = 0.046** η^2^ = 0.272	*p* = 0.267 η^2^ = 0.094	***p* = 0.001** η^2^ = 0.557	***p* = 0.012** η^2^ = 0.293	***p* = 0.011** η^2^ = 0.293	*p* = 0.733 η^2^ = 0.015	*p* = 0.641 η^2^ = 0.034
	NZBP	75 ± 5	74 ± 6	70 ± 6							
	CAFF	75 ± 7	77 ± 6	75 ± 6							
	NZBP + CAFF	72 ± 6	76 ± 4	72 ± 5							
Stroke Volume (mL)	PLA	68 ± 14	78 ± 11	72 ± 13	*p* = 0.793 η^2^ = 0.005	*p* = 0.592 η^2^ = 0.023	*p* = 0.961 η^2^ = 0.000	***p* = 0.004** η^2^ = 0.343	*p* = 0.929 η^2^ = 0.006	*p* = 0.106 η^2^ = 0.159	*p* = 0.051 η^2^ = 0.205
	NZBP	76 ± 18	73 ± 15	72 ± 16							
	CAFF	72 ± 16	75 ± 17	71 ± 15							
	NZBP + CAFF	73 ± 14	77 ± 18	73 ± 13							
Systemic Vascular Resistance (dynes⋅sec⋅cm^−5^)	PLA	2125 ± 470	1782 ± 341	1461 ± 332	*p* = 0.821 η^2^ = 0.004	*p* = 0.954 η^2^ = 0.000	*p* = 0.712 η^2^ = 0.011	***p* < 0.001** η^2^ = 0.583	*p* = 0.265 η^2^ = 0.097	*p* = 0.348 η^2^ = 0.077	*p* = 0.262 η^2^ = 0.098
	NZBP	1985 ± 698	1893 ± 466	1562 ± 536							
	CAFF	1968 ± 445	1884 ± 499	1620 ± 484							
	NZBP + CAFF	1951 ± 461	1956 ± 696	1522 ± 439							

PLA, Placebo; NZBP, New Zealand blackcurrant; CAFF, Caffeine; NZBP + CAFF, New Zealand blackcurrant + Caffeine. Values are mean ± SD. Data were analysed using factorial repeated-measures ANOVA with caffeine, NZBP, and time as within-participant factors. Significant effect of caffeine, NZBP, and time, and the caffeine × NZBP, caffeine × time, NZBP × time, and caffeine × NZBP × time interactions were assessed. Significant effects are shown in bold.

**Table A2:** Heart rate 1 h after consuming the study drink, during blocks 1 to 6 of the modified Loughborough Intermittent Shuttle Test, and 1 h after exercise.

		1 h After Drink	Block 1	Block 2	Block 3	Block 4	Block 5	Block 6	1 h After Exercise	Effect of Caffeine	Effect of NZBP	Effect of Caffeine × NZBP	Effect of Time	Effect of Caffeine × Time	Effect of NZBP × Time	Effect of Caffeine × NZBP × Time
Heart Rate (beats/min)	PLA	58 ± 4	152 ± 10	160 ± 7	161 ± 7	161 ± 7	157 ± 10	158 ± 8	78 ± 9	*p* = 0.785 η^2^ = 0.016	*p* = 0.520 η^2^ = 0.087	***p* = 0.015** η^2^ = 0.728	***p* < 0.001** η^2^ = 0.985	*p* = 0.873 η^2^ = 0.031	*p* = 0.572 η^2^ = 0.119	***p* = 0.032** η^2^ = 0.475
	NZBP	59 ± 7	152 ± 10	156 ± 8	156 ± 8	156 ± 8	153 ± 11	152 ± 13	74 ± 11							
	CAFF	59 ± 7	151 ± 15	158 ± 12	159 ± 9	157 ± 10	154 ± 9	155 ± 11	75 ± 9							
	NZBP + CAFF	58 ± 11	152 ± 13	162 ± 6	161 ± 6	160 ± 8	158 ± 9	158 ± 12	75 ± 12							

PLA, Placebo; NZBP, New Zealand blackcurrant; CAFF, Caffeine; NZBP + CAFF, New Zealand blackcurrant + Caffeine. Values are mean ± SD. Data were analysed using factorial repeated-measures ANOVA with caffeine, NZBP, and time as within-participant factors. Significant effect of caffeine, NZBP, and time, and the caffeine × NZBP, caffeine × time, NZBP × time, and caffeine × NZBP × time interactions were assessed. Significant effects are shown in bold.

### Appendix A.2 Perceptual Measures

**Table A3:** Perceptual measures: feeling scale and felt arousal scale at baseline, during blocks 1–6 of the modified Loughborough Intermittent Shuttle Test, and 1 h after completing the exercise.

		Baseline	Block 1	Block 2	Block 3	Block 4	Block 5	Block 6	1 h Post Exercise	Effect of Caffeine	Effect of NZBP	Effect of Caffeine × NZBP	Effect of Time	Effect of Caffeine × Time	Effect of NZBP × Time	Effect of Caffeine × NZBP × Time
Feeling Scale (−5 to +5)	PLA	1.9 ± 1.1	0.7 ± 2.2	−0.2 ± 1.7	−1.1 ± 1.8	−1.0 ± 1.8	−1.1 ± 2.2	−1.9 ± 2.3	1.6 ± 1.9	***p* = 0.006** η^2^ = 0.486	*p* = 0.886 η^2^ = 0.002	*p* = 0.858 η^2^ = 0.003	***p* < 0.001** η^2^ = 0.573	*p* = 0.067 η^2^ = 0.178	*p* = 0.260 η^2^ = 0.102	*p* = 0.254 η^2^ = 0.106
	NZBP	1.8 ± 2.0	0.1 ± 2.1	−0.4 ± 2.6	−1.1 ± 2.4	−1.2 ± 2.4	−1.2 ± 2.2	−0.7 ± 2.8	2.0 ± 2.1							
	CAFF	1.4 ± 1.4	1.2 ± 1.3	0.3 ± 1.5	−0.3 ± 1.5	−0.5 ± 2.3	−0.8 ± 2.6	−1.1 ± 2.7	2.2 ± 1.8							
	NZBP + CAFF	2.1 ± 1.3	1.3 ± 1.5	0.3 ± 1.3	0.1 ± 1.9	−1.0 ± 1.8	−0.9 ± 1.6	−0.9 ± 2.4	1.1 ± 1.8							
Felt Arousal Scale (1 to 6)	PLA	2.5 ± 1.0	3.9 ± 1.0	4.1 ± 1.1	4.1 ± 1.1	3.9 ± 1.2	4.0 ± 1.3	4.1 ± 1.3	2.6 ± 1.1	*p* = 0.747 η^2^ = 0.009	*p* = 0.853 η^2^ = 0.003	*p* = 0.097 η^2^ = 0.213	***p* < 0.001** η^2^ = 0.635	*p* = 0.408 η^2^ = 0.077	*p* = 0.406 η^2^ = 0.077	*p* = 0.384 η^2^ = 0.079
	NZBP	1.9 ± 0.8	4.0 ± 1.1	4.3 ± 1.2	4.3 ± 1.2	4.3 ± 1.2	4.4 ± 1.2	4.5 ± 1.0	2.9 ± 1.2							
	CAFF	2.6 ± 1.0	3.9 ± 0.8	4.0 ± 1.0	4.1 ± 1.2	4.5 ± 1.1	4.3 ± 1.2	4.4 ± 1.3	2.8 ± 1.1							
	NZBP + CAFF	2.23 ± 1.0	3.7 ± 1.0	4.0 ± 1.1	4.3 ± 1.0	4.3 ± 0.7	4.1 ± 1.0	3.6 ± 2.2	2.8 ± 1.0							

PLA, placebo powder; CAFF, caffeinated placebo powder; NZBP, New Zealand blackcurrant powder; NZBP + CAFF powder, caffeinated New Zealand blackcurrant powder. N/A: Not assessed. Values are mean ± SD. Data were analysed using factorial repeated-measures ANOVA with caffeine, NZBP, and time as within-participant factors. Significant effect of caffeine, NZBP, and time, and the caffeine × NZBP, caffeine × time, NZBP × time, and caffeine × NZBP × time interactions were assessed. Significant effects are shown in bold.

### Appendix A.3 Caffeine Metabolites

**Table A4:** Caffeine metabolite concentrations at baseline, 1 h after study drink consumption, and immediately after completing the modified Loughborough Intermittent Shuttle.

		Baseline	1 h After Consuming the Study Drink	1 h After Exercise	Effect of Caffeine	Effect of NZBP	Effect of Caffeine × NZBP	Effect of Time	Effect of Caffeine × Time	Effect of NZBP × Time	Effect of Caffeine × NZBP × Time
Paraxanthine (µg/mL)	PLA	0.31 ± 0.30	0.30 ± 0.24	0.31 ± 0.22	***p* < 0.001** η^2^ = 0.966	*p* = 0.061 η^2^ = 0.372	*p* = 0.612 η^2^ = 0.034	***p* < 0.001** η^2^ = 0.956	***p* < 0.001** η^2^ = 0.954	*p* = 0.309 η^2^ = 0.133	*p* = 0.577 η^2^ = 0.053
	NZBP	0.36 ± 0.32	0.37 ± 0.30	0.40 ± 0.20							
	CAFF	0.41 ± 0.30	2.39 ± 0.49	3.32 ± 0.66							
	NZBP + CAFF	0.56 ± 0.57	2.38 ± 0.84	3.19 ± 1.07							
Theophylline (µg/mL)	PLA	0.13 ± 0.02	0.12 ± 0.02	0.13 ± 0.02	***p* < 0.001** η^2^ = 0.781	*p* = 0.403 η^2^ = 0.071	*p* = 0.802 η^2^ = 0.007	***p* < 0.001** η^2^ = 0.722	***p* < 0.001** η^2^ = 0.747	*p* = 0.614 η^2^ = 0.045	*p* = 0.857 η^2^ = 0.015
	NZBP	0.12 ± 0.01	0.12 ± 0.01	0.12 ± 0.01							
	CAFF	0.12 ± 0.01	0.15 ± 0.02	0.17 ± 0.02							
	NZBP + CAFF	0.13 ± 0.02	0.15 ± 0.03	0.17 ± 0.03							
Caffeine (µg/mL)	PLA	0.14 ± 0.01	0.14 ± 0.01	0.14 ± 0.01	***p* < 0.001** η^2^ = 0.960	*p* = 0.274 η^2^ = 0.147	*p* = 0.300 η^2^ = 0.133	***p* < 0.001** η^2^ = 0.949	***p* < 0.001** η^2^ = 0.949	*p* = 0.334 η^2^ = 0.128	*p* = 0.320 η^2^ = 0.133
	NZBP	0.15 ± 0.02	0.15 ± 0.03	0.14 ± 0.03							
	CAFF	0.40 ± 0.84	3.39 ± 0.70	2.62 ± 0.56							
	NZBP + CAFF	0.51 ± 0.87	3.29 ± 0.69	2.43 ± 0.85							

PLA, Placebo; NZBP, New Zealand blackcurrant; CAFF, Caffeine; NZBP + CAFF, New Zealand blackcurrant + Caffeine. Values are mean ± SD. Data were analysed using factorial repeated-measures ANOVA with caffeine, NZBP, and time as within-participant factors. Significant effect of caffeine, NZBP, and time, and the caffeine × NZBP, caffeine × time, NZBP × time, and caffeine × NZBP × time interactions were assessed. Significant effects are shown in bold.
